# Nuclear DNA-Content in Mesenchymal Lesions in Dogs: Its Value as Marker of Malignancy and Extent of Genomic Instability

**DOI:** 10.3390/cancers4041300

**Published:** 2012-12-03

**Authors:** Kim M. Boerkamp, Gerard R. Rutteman, Marja J. L. Kik, Jolle Kirpensteijn, Christoph Schulze, Guy C. M. Grinwis

**Affiliations:** 1 Department of Clinical Science of Companion Animals, Faculty of Veterinary Medicine, UU, Yalelaan 104, 3584 CM, Utrecht, The Netherlands; E-Mails: G.R.Rutteman@uu.nl (G.R.R.); J.Kirpensteijn@uu.nl (J.K.); 2 Department of Pathobiology, Faculty of Veterinary Medicine, UU, Yalelaan 1, 3508 TD, Utrecht, The Netherlands; E-Mails: M.Kik@uu.nl (M.J.L.K.); Christoph.Schulze@Landeslabor-bbb.de (C.S.); Grinwis@uu.nl (G.C.M.G.)

**Keywords:** aneuploidy evolution, canine, sarcomas, DNA index

## Abstract

DNA-aneuploidy may reflect the malignant nature of mesenchymal proliferations and herald gross genomic instability as a mechanistic factor in tumor genesis. DNA-ploidy and -index were determined by flow cytometry in canine inflammatory or neoplastic mesenchymal tissues and related to clinico-pathological features, biological behavior and p53 gene mutational status. Half of all sarcomas were aneuploid. Benign mesenchymal neoplasms were rarely aneuploid and inflammatory lesions not at all. The aneuploidy rate was comparable to that reported for human sarcomas with significant variation amongst subtypes. DNA-ploidy status in canines lacked a relation with histological grade of malignancy, in contrast to human sarcomas. While aneuploidy was related to the development of metastases in soft tissue sarcomas it was not in osteosarcomas. No relation amongst sarcomas was found between ploidy status and presence of P53 gene mutations. Heterogeneity of the DNA index between primary and metastatic sarcoma sites was present in half of the cases examined. Hypoploidy is more common in canine sarcomas and hyperploid cases have less deviation of the DNA index than human sarcomas. The variation in the presence and extent of aneuploidy amongst sarcoma subtypes indicates variation in genomic instability. This study strengthens the concept of interspecies variation in the evolution of gross chromosomal aberrations during cancer development.

## Abbreviations

CKC: Canine Kidney Cells
CRBC: Chicken Red Blood Cells
CV: Coefficient of Variation
DI: DNA Index
FCM: Flow Cytometry
HS: Histiocytic Sarcoma
MTB: Malignant Tumor of Bone
OS: Osteosarcoma
PD: Peridiploid
STS: Soft Tissue Sarcoma

## 1. Introduction

In dogs cancer is the most common cause of non-traumatic death [1]. Several types of canine malignant neoplasms—based upon pathobiology—can serve as useful models for rare cancers in humans, including osteosarcomas (OS) and soft tissue sarcomas (STS) [2,3,4], both types being relatively common in the dog [5,6].

As in humans, the prognosis of canine tumor patients is related to tumor location and, for malignant tumors, clinical stage [7]. A key factor in this assessment is the histological phenotype including malignancy grade [4,8,9], but there is a significant morphological overlap between malignant, benign, and reactive mesenchymal lesions in the human [10,11]. For both species, classification and management of mesenchymal tumors is complicated by the fact that these form a very heterogeneous group [9,12]. In order to improve the prognostic value of histological examination of Malignant Tumor of Bone (MTB) and of STS, several classification and grading systems have been established in humans [13], and later adopted for use in the dog [8,9], but the prognostic value of these systems for many individual tumors is limited [4,9,14]. Therefore, objective prognostic criteria are urgently needed.

Determination of the DNA-ploidy status may help to discriminate non-neoplastic or benign neoplastic lesions from (pre)malignant neoplastic conditions [15], including lesions of mesenchymal origin [16,17,18,19]. In humans, DNA-aneuploidy is more common in high-grade sarcomas than in those of low- or intermediate-grade [16,18,20,21]. These potential discriminatory values still need to be examined in dogs. In addition, DNA-aneuploidy has been reported to have prognostic value in several cancer types in humans [22,23,24]. However, in some sarcoma subtypes, this is not always the case [16,21,25,26,27,28,29,30,31,32].

Besides the diagnostic value that the DNA-ploidy status can have, the DNA index (DI) distribution may help to comprehend the nature of genomic changes during tumorigenesis [33,34,35]. It reflects gross chromosomal changes [36] that, as already hypothesized one century ago [37], appear to play a mechanistic role in tumorigenesis, the extent of which is subject of debate [33,34,35,36,38,39,40,41,42,43,44,45,46,47,48,49]. In approximately two-thirds of human malignant solid cancers, the evolution of karyotypic alterations over many cell divisions until cancer clinically manifests, is thought to be reflected by the increase of total nuclear DNA content, which peaks at 1.6-fold of the normal amount [33,34,35,50]. In the other one-third of human cancers, the deviation of the DI is much less prominent, or cannot be discriminated from normal due to only minute or balanced chromosomal changes [15]. In general, a significant decrease in the DNA-content or DNA-hypoploidy is rare in most human solid cancers, with a few exceptions such as chondrosarcomas [51].

Loss of function of the *P53* pathway has been hypothesized to be one important factor in the development of aneuploid cancers [52]. As in humans, most cancers in dogs, such as thyroid and mammary carcinomas, are DNA-aneuploid, but the extent of the aberration of the DI in DNA-aneuploid cancers is less and DNA-hypoploidy more common in such carcinomas in the dog than in humans [53,54,55]. In fact, these observations led us to hypothesize that there is an interspecies evolutionary variation in the manifestation of DNA-aneuploidy in tumors, when comparing humans and dogs [44].

In continuation with our earlier research [53,54,55], we now examined the DNA-ploidy distribution pattern as determined by flow cytometry (FCM) in a series of fresh frozen samples of canine benign and malignant mesenchymal lesions. Preliminary results of this study have been presented as poster at the ESF conference (ESF Conference, Dresden, March 2010). For sarcomas, the data were compared with information on clinical stage, histological subtype and grade and the mutational status of the *P53* gene, and with published data in human sarcomas. Comparison of observations in the current and earlier studies on canine neoplasms to those published on human cancers, points to interspecies variation in the driving force of aneuploidy in the development of malignant tumors.

## 2. Results

### 2.1. DNA-Ploidy Status in Primary Lesions

**Figure 1:**
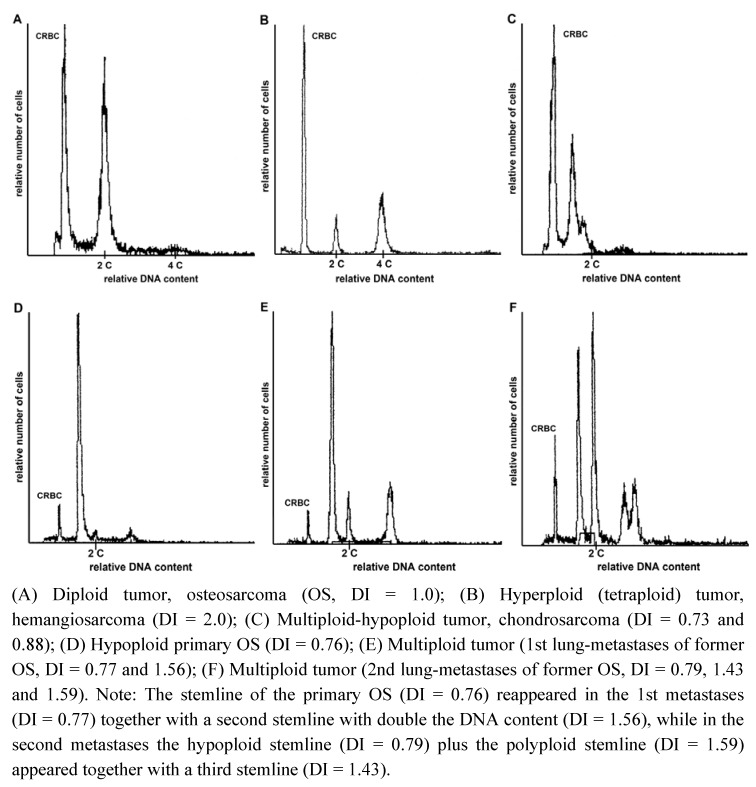
Example of DNA content histograms of canine tumors.

The arithmetical mean of the CV of G_0__–1_ populations of all lesions was 3.38 (1.4–5.0). The DNA ratio of the non-neoplastic G_0__–1_ diploid cells as compared to CRBC G_0__–1_ cells fell within the margins established in earlier studies [53,56,57]. The ploidy status as defined by FCM-base histograms (Figure 1) was normal (diploid) in all non-malignant lesions except one lipoma with a small (10% of all nuclei) aneuploid peak (DI 1.20).

**Figure 2:**
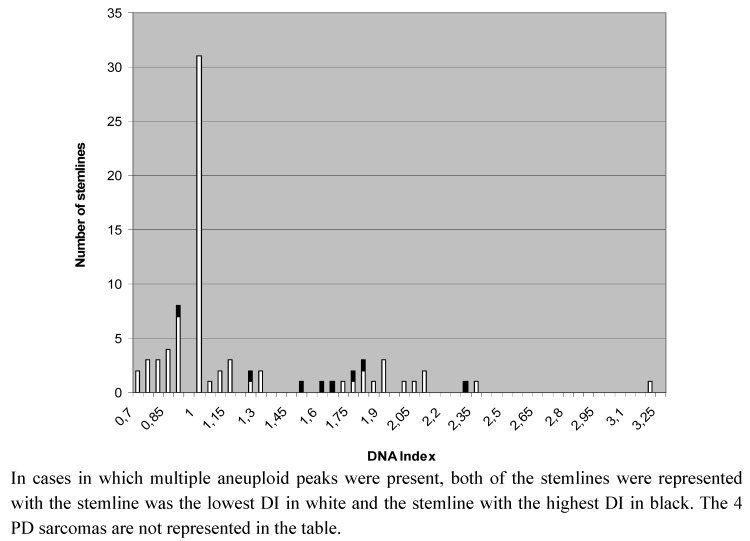
DNA indices of stemlines present in all 77 primary malignant lesions.

In three dogs with histiocytic sarcoma (HS), there was multi-organ involvement. To attribute a ploidy status in such cases, the DI of the largest of multiple tumors (one dog) or one out of multiple equally-sized lesions with equal DI-results was used in the categorization of primary malignancies. There were 42 aneuploid cases out of 77 primary malignant tumors (55%), and four (5%) PD cases. We found a significant difference in the ploidy-status of primary malignancies compared to benign neoplastic lesions (*p* = 0.023) and to non-neoplastic proliferative lesions (*p* = 0.0005). Out of all 43 aneuploid primary tumors (including one lipoma), 35 had only a single aneuploid G_0__–1_ population named stemline (81.4%), whereas eight (18.6%) had multiple aneuploid stemlines. The distribution of the DIs of all stemlines in primary sarcomas is shown in Figure 2. The ploidy status did not vary (*p* = 0.62) between all STS as compared to all MTB.

Amongst STS, malignant peripheral nerve sheath tumors (MPNST) and leiomyosarcomas had a significantly lower rate of aneuploidy (Table 1) as compared to all other STS (*p* = 0.003 and 0.03, respectively). In contrast, HS and synovial cell sarcomas had an increased rate of aneuploidy when compared to all other STS (*p* = 0.01 and 0.02, respectively) or to MPNST or leiomyosarcomas (*p* < 0.02).

Twenty of the 77 primary sarcomas had hypoploid stemlines (26%, or 48% of the aneuploid cases). Amongst aneuploid sarcomas the occurrence of hypoploidy in STS (9 of 25; 36%) and MTB (11 of 17; 65%) did not differ significantly (*p* = 0.115).

Noteworthy is the relative high DI within the three liposarcoma cases (2.0, 2.3 and 3.2 respectively), in relation to the general DI distribution in primary malignancies (Figure 1). There was no significant relation between ploidy status and histological malignancy grade (Table 2, grade I + II *versus* III in MTB: *p* = 1.0, in STS: *p* = 0.16, in all sarcomas: *p* = 0.15). Note that this analysis excluded the three PD cases. Examination of a possible relation between ploidy-status and the p53 gene mutational status in 44 sarcomas was negative (Table 3).

**Table 1:** DNA-ploidy status in 77 sarcomas according to subtype.

Subtype	Diploid (n)	Aneuploid (n)	Peridiploid (n)	Total (n)
**Malignant tumors of bone**				
Osteosarcoma	9	14	3	26
Chondrosarcoma	1	2	-	3
Multilobular tumor of bone	0	1	-	1
**Total**	**10**	**17**	**3**	**30**
**Soft tissue sarcomas**				
Fibrosarcoma	2	1	-	3
Sarcoma-not otherwise specified	3	1	-	4
Rhabdomyosarcoma	2	3	-	5
Malignant peripheral nerve sheath tumors	7	1	-	8
Synovial sarcoma	0	5	-	5
Liposarcoma	0	3	-	3
Leiomyosarcoma	6	2	-	8
Hemangiosarcoma	1	3	-	4
Histiocytic sarcoma	0	6	1	7
**Total**	**21**	**25**	**1**	**47**

**Table 2:** DNA-ploidy status and histological malignancy grade in 59 sarcomas.

Tumor type and grade	Diploid (n)	Peridiploid (n)	Aneuploid (n)
**Malignant tumor of bone**			
-grade I	0	0	0
-grade II	1	1	2
-grade III	8	2	12
**Soft tissue sarcoma**			
-grade I	5	0	2
-grade II	5	0	2
-grade III	8	0	11

**Table 3:** Presence of p53 mutations in sarcomas (n = 44) as related to the ploidy-status.

	Diploid	Aneuploid	Peridiploid
P53-wt	12	14	2
P53- alteration	7	9	1

Note: only mutations predicted to alter the amino acid composition of the p53 protein were counted.

We then compared ploidy status with metastatic behavior. Only cases with macroscopic metastasis were considered, found either at first presentation (including those with postmortem after euthanasia) or during a follow up period for up to one year. PD cases (n = 4) and cases with less than one year follow up after tumor resection (n = 22, including three PD cases) were excluded. In the category of MTB only 20 dogs with OS fulfilled the criteria; all developed metastases either at first presentation (n = 2) or after surgical removal of the primary tumor (n = 18) without any influence of ploidy status (Table 4).

**Table 4:** The metastatic behavior of the sarcomas and the ploidy status of the primary lesion.

Tumor group	Ploidy status	
	Diploid	Aneuploid
**Osteosarcomas**		
-Metastases	8	12
-No metastases	0	0
**Soft tissue sarcomas **		
-Metastases	5	17
-No metastases	8	3

Note: PD cases were excluded, as well as cases that lacked information on metastatic growth or recurrence within the first year following the initial diagnosis.

When viewing STS as a group (including HS), 22 out of 34 dogs had metastases at first presentation or during follow up after tumor resection. Aneuploid STS more often had metastases than diploid STS (*p* = 0.0092). The difference was no longer significant (*p* = 0.067) after exclusion of HS. All HS cases but one (which was euthanized upon diagnosis) had metastases at first presentation.

### 2.2. Heterogeneity of Ploidy Status or DI

In 12 dogs, we were able to compare the DI of the primary and metastatic lesions (Table 5). In six of these dogs, a significant variation in DI was noticed: The DI changed from a diploid into an aneuploid (hyperdiploid) pattern in two sarcomas and from an aneuploid (hyperdiploid) to a diploid pattern in one other. In one dog with HS, in which the largest tumor (lung) and a sternal lymph node were both analyzed, the pulmonary lesion was PD and the nodal was low-level hypodiploid (DI 0.93). No particular significance was ascribed to this difference. In another three cases extra stemlines were identified on one location as compared to the other location(s), which in two of these cases led to the (dis)appearance of a stemline, containing twice the total DNA content.

**Table 5:** Comparison of the DNA index (DI) in primary *versus* metastatic lesions (Met. 1 to 4) from the same tumors.

Case	Primary	Met. 1	Met. 2	Met. 3	Met. 4
Osteosarcoma 1 *	2.10	1.10/2.14	1.12		
Osteosarcoma 2 *	0.76	0.77/1.56	0.79/1.43/1.59	0.79/1.55	0.78/1.55
Osteosarcoma 3 *	1.0	1.88			
Osteosarcoma 4	1.0	1.0	1.0		
Synovial cell sarcoma *	0.89/1.80	1.77			
Hemangiosarcoma *	1.89	1.0	1.0		
Sarcoma-NOS *	1.0	1.77			
Fibrosarcoma	1.0	1.0	1.0		
Malignant Peripheral Nerve Sheet Tumor	1.0	1.0			
Synovial Cell Sarcoma	0.72	0.72			
Histiocytic Sarcoma	PD	0.93			
Liposarcoma	2.03	1.97			

Note: The variations in the DI became visible as peaks with >10% of the total cell population analyzed. Met: Metastasis, NOS: Not otherwise specified. Marked with an asterisk (*) are six dogs that had significant variation in DI comparing primary-and metastatic lesion.

## 3. Discussion

Our study indicates that the existence of aneuploidy in canine mesenchymal proliferative lesions is suggestive but no proof of a malignancy, in accordance with observations in humans [16,58,59,60], although it must be recognized that a diploid status does not rule out malignancy. This finding may lead to additional cytological/histological testing of canine mesenchymal lesions in which the results of routine diagnosis remain ambiguous.

For sarcomas in humans, a relationship between histological grade of malignancy and ploidy pattern has often been reported [16,20,59,61], but no such relationship was evident in our study. In part, this may be related to the relatively high frequency of hypoploid cancers, and the related presence of smaller nuclei, which might be judged lower grade by pathologists.

As in earlier studies in mammary malignant tumors in dogs [22,53,55] and in humans [22,23,30], we were also able to demonstrate a relation between ploidy status and risk of metastases in STS. Many studies in humans [28,30,31,62] reported a similar correlation, although this is not universal [58]. Striking was the absolute lack of a relation between ploidy status and risk of metastasis in OS, which is in contrast to OS in humans [32,63]. In diploid cases within particular types of cancer more detailed cytogenetic analyses are required to discern those changes that predict metastatic behavior [62,64].

Consistent with early research [65,66,67], clear similarities were found with respect to aneuploidy occurrence amongst STS (53%) and MTB (57%) when compared to such sarcomas in humans [50,58,60]. For the subtypes of MPNST and leiomyosarcoma a diploid status is common in both dogs (as seen in our study) and humans [68]. However, in most human sarcomas hypodiploidy is rare [10,50], with the highest reported rate being 11% [58], while chondrosarcomas are an exception since they are frequently hypoploid [69]. We observed a remarkable overall hypoploidy incidence of 26% (48% of aneuploid cases). One other study in canine OS reported a somewhat lower figure [67]. In addition, both studies indicate that for many subtypes of canine sarcomas the net increase in DNA content in hyperploid cases is modest when compared to their human counterparts [16,58,70] albeit that some sarcoma types, such as the three liposarcomas in our study, form an exception, since these were all (hyper)tetraploid.

Since similar observations have been made in other types of cancer in the dog such as thyroid and mammary carcinomas and malignant lymphomas [53,54,56,57], it seems that the dog is particularly prone to the development of aneuploid tumors associated with either chromosome loss or low number chromosome gain, and less frequently to hypertriploid tumors that are common in humans [33,34,40,50]. This feature seems at variance with ploidy evolution patterns described in humans. In humans, this evolution can be divided in stages with early on the development of tetraploidy which is followed by chromosome loss and later on leads to a major proportion (amongst aneuploid cases) of cancers manifesting an increase in DI of approximately 1.6–1.7 [33,34,35,40,45,50]. Still, in a few sarcomas of the current study and in mammary carcinomas [53,65] tetraploidization (in the primary cancer) followed by an appearance of hypotetraploid stemlines (in its metastases) has been observed. A possible explanation for this difference in ploidy evolution could be the presence of a more powerful defense mechanism in humans against the tumorigenic effects of hypodiploidy or low level hyperploidy [33,71]. While for many human cancers a greater destabilization over multiple phases of destabilizing events seems necessary to arrive at a fully malignant state, such state may be reached with less destabilization in the dog, as hypothesized earlier [44]. How some cancers can reach at a fully malignant state while remaining DNA-diploid is uncertain. More subtle and sometimes balanced chromosomal gains and losses have been observed by cytogenetic analysis in some such human cancers [34] including sarcomas [19,68], and also in canine sarcomas [72,73] and carcinomas [74]. In other human diploid cancers however, structural chromosomal abnormalities are absent, and the transformation to malignancy seems to be driven by defects in DNA repair pathways leading to microsatellite instability, with microsatellite instability and aneuploidy being mutually exclusive phenomena [46].

It must be recognized that the karyotypic alterations that occur in dog cancer are to some extent at variance with those described for human cancers. The autosomes in normal dog cells are acrocentric/telocentric, and changes in canine cancers often concern centric fusions [74,75,76,77] albeit that alterations such as trisomies or monosomies and translocations are also frequent [72,75,78,79] as well as smaller structural abnormalities that have been detected with comparative genomic hybridization studies [73,79,80,81] in the past few years. Still, as mentioned in an extensive review by Breen and Thomas, “tumors of the same histological types in both species present with equivalent cytogenetic lesions” [82].

In cancers that harbour significant chromosomal alterations, even if not recognized as DNA-aneuploid, the cause underlying this chromosomal instability has been subject of study and aberrations in many pathways, in particular those related to sister chromatid cohesion and segregation have been suggested as possible causes [46,47,49,83,84,85]. Contrasting views exist as to whether loss of *P53* function is essential for the development of aneuploidy [52,86,87,88]. In the current study, no relation between *P53* mutations and DNA-ploidy status was found. Although it must be recognized that analysis for *P53* mutations concerns only part of the many elements involved in the *P53* pathway, our results are in clear contrast with a study in human sarcomas that demonstrated a relation between the presence of *P53* mutations and DNA-aneuploidy [61].

Regarding whether and how aneuploidy may be essential for the development of cancer [38] it is essential to agree on the definition of malignancy. Most oncologists agree that proof of metastatic potential is not the only prerequisite for a tumor to be considered malignant and that tumors with extensive infiltrative destructive growth and low or late risk of metastases such as leiomyosarcomas and MPNSTs in the dog should also be regarded as malignant [7]. In our study, these sarcomas were only rarely found to be aneuploid. There is no doubt that many malignancies with full malignant potential are aneuploid, reflecting gross quantitative genomic destabilization. Several major disturbances must have taken place in order to overcome restraints, which are—in part tissue specific—against malignant transformation. Other cancers, such as the diploid OS in the current study, can reach this state of progression with much less genome destabilization. Functional changes that may allow malignant behavior in such cancers may include *P53* inactivation or *MDM2* amplification [89,90] as well as many other genetic alterations. Such changes may be called high hierarchy changes. This is opposite to many more subtle low hierarchy changes that can accumulate during progressive genomic destabilization and, by chance or number, can also disrupt crucial control mechanisms. To some extent and for some types of sarcomas (such as MPNST and leiomyosarcoma), a diploid status is then related to low metastatic potential. For OS in the dog a highly metastatic state can be either reached by appearance of a relatively low number of high hierarchy genetic changes, and low level of genomic destabilization in the form of chromosomal instability, while in others a high level of genomic destabilization, involving more lower hierarchy changes, may lead to this state.

The debate on gene mutation *versus* aneuploidization as cause of cancer cannot be resolved, since both phenomena may be linked and cooperative in tumor development [36,48].

Once a malignant tumor has developed, the same DI can often be found in both the primary and the metastatic lesion. Sometimes, haploidization or duplication of such stemlines occurs, with variation between metastases. In other tumors in the current and earlier studies [53,65,67] stemlines were found which really looked like an unrelated clone, able to propagate as independent subpopulation, as hypothesized previously [91].

## 4. Experimental Section

### 4.1. Animals

Lesions from pet-dogs of various breeds, sex and age were selected for this study. Samples with suspicion of a neoplastic origin were obtained, with informed consent from the owner, by biopsy or surgical excision as part of normal diagnostic procedure or treatment, or at postmortem immediately following euthanasia. After previous studies by our research group on DNA-ploidy* of carcinomas [53,54,56] and of malignant lymphomas [57], the focus was on proliferative, inflammatory or neoplastic lesions of the mesenchyme. 

All samples had been characterized by routine histopathology by different veterinary pathologists and were centrally reviewed for the current study. The ploidy assessment was not possible in three soft tissue sarcomas, one inflammatory lesion and two benign lesions, probably due to the amount of debris present. These samples were excluded from further analyses. Remaining samples, from 95 dogs, included non-neoplastic, proliferative, inflammatory lesions (n = 10), benign tumors (n = 8: lipoma 4), fibrous dysplasia of bone, osteochondroma, leiomyoma, fibrous epulis), and 77 malignancies (including one local recurrence; detailed description is listed in Table 2), from 77 dogs. From 12 of these 77 dogs both primary and recurrent specimens were available for examination.

Prior to collection of the tissue samples, none of the patients had received any cytostatic or radiotherapeutic treatment. Data on the occurrence of metastasis at first presentation or for up to one year after surgery were collected from the hospital records. Where in the following text the word ploidy is used as related to the current study, it indicates DNA-ploidy.

### 4.2. Preparation of Samples

Upon surgery or euthanasia, tissue samples were immediately placed in melting ice. From areas of the mass not hampering full histological evaluation, thus avoiding planes of resection, blocks of approximately 5 mm^3^ were cut, trimmed of fat and necrotic parts, snap-frozen in liquid nitrogen and stored at −70 °C until further analysis. The tissue samples were thawed only once, directly prior to FCM. Adjacent blocks including the resection planes were fixed in neutral phosphate-buffered 10% formalin for histological examination.

### 4.3. Histological Examination

The tissues were routinely processed; paraffin embedded and cut to 4–6 µm sections that were stained with hematoxylin-eosin (H&E). Cases were re-evaluated by board-certified veterinary pathologist (CS, GCMG) and categorized according to the WHO classification for tumors in domestic animals (WHO 1994, 1998). STS were grouped by their entity, including additional immunohistochemistry if deemed appropriate [89,92,93]. Most cases were reviewed, except for eleven due to sample loss, for which the original diagnoses were used. With the exclusion of histiocytic sarcomas (HS), a proper grading system is available for STS [8,9] as well as for OS [4]. This was applied for the current series, except for ten cases that could not be graded leaving a total of 59 cases.

### 4.4. Flow Cytometry Analysis of Nuclear DNA Content

FCM of nuclear DNA content was performed as previously described [53,57] using the detergent-trypsin procedure of Vindolov *et al*. [94]. Isolated nuclei from all lesions were stained with propidium iodide (Sigma Chemical Co., St. Louis, MO, USA). All neoplastic samples were required to contain an adequate proportion of at least 20% tumor cells and the cell yield of all specimens had to permit analysis of ≥5,000 cells in each assay. As external standard, Chicken Red Blood Cells (CRBC) was added to determine the position of the G_0__–1_ peak(s). In some samples two G_0__–1_ peaks were discerned of which the DNA content both fell within the range of the ratio of the DNA content of normal dog diploid cells compared to that of CRBC. Then a second analysis was done with and without addition of Canine Kidney Cells (CKC) as a diploid standard. The increase in height of one of those peaks upon addition of CKC identified this peak as diploid and the other as aneuploid. The ploidy analysis itself was performed using the FAC Scan 3 flow cytometer (Becton Dickinson, Mountain View, CA, USA). The device is able to detect changes above 5% in nuclear DNA content. The propidium iodide fluorescence was excited at 488 nm and measured at 585 nm. Since past research has indicated that, by measuring only a single tissue block, true aneuploid stemlines can sometimes be missed [58], many lesions—in particular large-sized ones—were analyzed using two or more tissue blocks. In 11 primary tumors of larger size (>3 cm) more than one tissue block was analyzed to assess possible intra-tumor heterogeneity. When a difference was found between the separate analyses, the measurement was repeated several times with and without the external CKC reference standard. 

### 4.5. DNA-Ploidy Assessment

The DI is defined as the ratio of the modal channel number of the G_0__–1_ peak of the (neoplastic) cell population in relation to the modal channel number of the G_0__–1_ fraction of diploid cells. The latter was recognized by its relative position to the G_0__–1_ peak of CRBC and by is position to the normal CKC (normal dog reference) peak [53]. A sample was considered diploid if the DI was between 0.95 and 1.05 and aneuploid if there was a distinct G_0__–1_ population with DI < 0.95 or > 1.05. A peak with a DI of 1.90–2.10 was considered to be a tetraploid G_0__–1_ peak if it contained >20% of the total number of analyzed cells and if a G2-M peak was also present. Aneuploid stemlines were subdivided into hypoploid (DI < 1.0) or hyperploid (DI > 1.0) When more than one aneuploid G_0__–1_ population was present the sample was classified as multiploid. For all samples, the coefficient of variation (CV) for the G_0__–1_ peak was measured. A CV above 5% was considered suspicious for the presence of an aneuploid peak within a diploid population. Samples with peaks with a CV > 5.1% were re-analyzed with and without the CKC reference cells, and if this led to a clear recognition of two separate peaks, it was considered aneuploid. However, if under these conditions a single G_0__–1_ peak with CV > 5.1% remained, the sample was considered neither diploid nor aneuploid but peridiploid (PD). All non-diploid, non-PD lesions were grouped together as aneuploid, including those with heterogeneity in ploidy status within the primary site.

### 4.6. Mutational Analysis of the p53 Gene

In two earlier studies, part of the sarcomas had been analyzed for the presence of p53 mutations. For STS the p53 gene was analyzed for exons IV–VIII, covering most of the mutations occurring in cancer [89]; for MTB exons I-X were examined [90]. Eight STS had been analyzed during the study of MTB that have not yet been published. The results of both investigations including a total of 44 sarcomas were used for a comparison with the ploidy-status. Only mutations predicted to affect the amino-acid sequence of the p53 gene [95] including point mutations, deletions and insertions, and mutations of the splice site were counted.

### 4.7. Statistical Analysis

Differences in frequency distribution in data groups were analyzed using Fishers exact test. The level of significance was set at 0.05. PD samples were excluded in the statistical analysis comparing diploid and aneuploid status.

## 5. Conclusions

In conclusion, aneuploidy is frequent in various canine malignant lesions, with prominent variation amongst the different sarcoma types. The overall frequency of aneuploidy is largely in accordance with findings in humans [50]. However, the high frequency of tumors with hypoploidy and low-level hyperploidy in various canine cancers [53,54,57] when compared to human malignancies including sarcomas [50], is striking. In human solid cancers hypoploidy is rare and the majority of cases are hypertriploid thought to arise from a tetraploid intermediate (33–35, 46, 50). Our findings therefore are suggestive of interspecies variation in aneuploidy evolution and genomic destabilization during carcinogenesis.
